# Identification of a novel loss-of-function mutation of the *GLA* gene in a Chinese Han family with Fabry disease

**DOI:** 10.1186/s12881-018-0734-2

**Published:** 2018-12-27

**Authors:** Chi Zhou, Jin Huang, Guanglin Cui, Hesong Zeng, Dao Wen Wang, Qiang Zhou

**Affiliations:** 1 Division of Cardiology, Department of Internal Medicine, Tongji Hospital, Tongji Medical College of Huazhong University of Science and Technology, Hubei Key Laboratory of Genetics and Molecular Mechanisms of Cardiological Disorders, Wuhan, 430030 China; 2 Division of Hematology, Department of Internal Medicine, Tongji Hospital, Tongji Medical College of Huazhong University of Science and Technology, Wuhan, 430030 China

**Keywords:** Fabry disease, Genetics, α-Gal a, DGJ

## Abstract

**Background:**

Fabry disease is an X-linked recessive lysosomal disorder caused by deficient enzymatic activity of α-galactosidase A (α-Gal A). The insufficient enzymatic activity leads to excessive accumulation of glycosphingolipids, the substrates of the enzyme, in lysosomes in organs and tissues. Mutations in the α-Gal A gene (*GLA*, Xq22) have been proven to be responsible for Fabry disease.

**Methods:**

In this study, we report a four-generation pedigree with left ventricular hypertrophy and chronic renal failure that was diagnosed by sequencing the *GLA* gene. An over expression system was constructed to evaluate the function of the detected mutation.

**Results:**

We identified a novel mutation in exon 6 of the *GLA* gene, p.Asn278Lys, which completely co-segregated with the disease phenotype. The protein level of α-Gal A was significantly lower in the variant group than in the wild-type group; additionally, the pharmacological chaperone 1-deoxy-galactonojirimycin (DGJ) effectively normalized the enzyme activity of α-Gal A and its decline at the protein level.

**Conclusions:**

This study is the first to report a novel loss-of-function mutation, p.Asn278Lys, in exon 6 of the *GLA* gene as a genetic aetiology for Fabry disease. In addition, we analysed the feasibility of DGJ as a therapeutic approach for this particular *GLA* mutation.

## Background

Fabry disease (FD, OMIM: 301500) is an X-linked inherited lysosomal disorder caused by the deficient activity of the lysosomal enzyme α-galactosidase A (α-Gal A). In males, the diagnosis of Fabry disease can be made easily by measuring enzyme activity in leukocytes. This disease was first independently described by two dermatologists, Johannes Fabry and William Anderson, in 1898 [1, 2]. The reported prevalence of FD is approximately 1:40,000 individuals worldwide [3], but new-born screening for FD has shown that the incidence is up to 1 in 3000 or even 1 in 1250 male births [4]. The incidence of FD is underestimated due to the existence of milder, later-onset variants and misdiagnosis as multiple organ involvement [5, 6].

The lysosomal enzyme α-Gal A catalyses the removal of galactose from oligosaccharides, glycolipids, and glycoproteins during the catabolism of macromolecules. [3]. Deficient enzymatic activity of α-Gal A induces the progressive intra-lysosomal deposition of glycosphingolipids, predominantly globotriaosylceramide in plasma, cardiomyocytes, vascular endotheliocytes, dorsal root ganglia neuronal cells, and a range of renal cell types [7]. The classic phenotype of FD involves acroparaesthesia and angiokeratomas in youth and progressive organ dysfunction in the kidney, heart, and nervous system [8]. Many symptoms of FD are also observed in other diseases, such as cerebrovascular disease and cardiovascular disease, which often leads to the misdiagnoses of this disease, especially the variant form. A considerable number of female patients do not exhibit abnormal enzyme activity, which makes diagnosis more complicated. Female heterozygotes must be diagnosed by genetic analysis, regardless of enzyme activity.

The genetic aetiology of FD is useful for a definitive diagnosis. The *GLA* gene encoding α-Gal A is located on the long arm of the X chromosome (Xq22.1) is 12 kb in length, with 7 exons. Hundreds of mutations in G*LA,* including missense and nonsense mutations and splicing defects, have been associated with FD, most of which are unique (“private mutations”) in each novel proband [9–11]. The pharmacological chaperone Migalastat was approved only recently, first by the EMA in Europe and then by the FDA, whereas enzyme replacement therapy (ERT) has effectively been available since 2001. These treatments have been shown to improve health-related quality of life [3, 12–14]. Early initiation of ERT prior to irreversible organ dysfunction is important, as the effects of improving cardiac function and reducing symptoms are minimal if the treatment is not started early enough [15–17].

To achieve a precise diagnosis and reverse the development of the disease in the early stage, genetic testing of the whole *GLA* gene and the flanking regions is strongly recommended. In the present study, we report a four-generation pedigree mainly presenting as chronic renal dysfunction but without a clear diagnosis at the time. We reached the diagnosis of FD by sequencing the whole exon regions of the *GLA* gene and subsequent functional analysis in an over-expression cell system.

## Methods

### Subjects

We report a four-generation family comprising 51 members (28 males, 23 females, 4 people of them died). The proband was a 65-year-old male patient. Seven of his family members were also included in this study. The patient underwent thorough physical examinations and other tests, including urine protein tests, electrocardiography, echocardiography, cardiac magnetic resonance, α-Gal A enzyme activity testing, genetic testing and skin biopsy. The family members were subjected only to a review of their medical histories, physical examination and genetic testing. The clinical features of the other patients in this pedigree were dictated by the proband, and the family members were enrolled in the present study. A total of 300 ethnically matched controls were used to detect the allele frequency of the identified variant in the normal population. Written information consent for participation and publication were obtained from all the individuals which are described in this manuscript. The patient in Fig. 2a consented to the inclusion of his picture in this manuscript in written format. The parents provided consent in the case of children under 16. The present study was approved by the Medical Ethics Committee of Tongji Hospital, Tongji Medical Collage, Huazhong University of Science and Technology, and complied with the principles of the Declaration of Helsinki.

### DNA isolation and DNA sequencing

Peripheral blood samples were collected from the patient, seven of his family members, and normal controls by using EDTA-K2 tubes. Genomic DNA was extracted by using the AP-96-BL-GDNA-12 kit (*AXYGEN BIOSCIENCES*, California, USA) according to the manufacturer’s instructions and stored at − 80 °C until further use. All seven exons and adjacent noncoding regions were amplified by PCR and subsequently screened via directional Sanger sequencing with the ABI 3130 BigDye Terminator v3.1 Cycle Sequencing Kit (Applied Biosystems, USA) on an ABI 3130xl sequencer. The primer sequences and amplicon sizes are shown in Table 1. The sequence analyses were performed by using a 3130 Genetic Analyzer (Applied Biosystems, USA). Genetic screenings of the mutation sites were conducted for 300 local control subjects.

**Table 1:** Primer sequences and amplicon sizes for *GLA* gene sequencing

Exon	Sense primer	Anti-sense primer	Amplicon size (bp)
GLA-E1	5’ CGTGACTGATTATTGGTCTACCTCTG 3’	5’ CGTTGAGACTCTCCAGTTCCCC 3’	433
GLA-E2	5’ TAACGGGATAAGAGAGACAAAAGAAA 3’	5’ AACTCTTGACCTCAGGTGATCCA 3’	521
GLA-E3	5’ AATACCTGGTGAAGTAACCTTGTCTC 3’	5’ CTTTCCTTTGTGGCTAAATCTCTG 3’	329
GLA-E4	5’ CCCTGGATGACAGACTGAACCC 3’	5’ GGAGACCTTGGTTTCCTTTGTTGT 3’	293
GLA-E5	5’ TCAATCTGTAAACTCAAGAGAAGGC 3’	5’ TCACATAAAGCCTCCTCCCAG 3’	375
GLA-E6	5’ CAGGATGCTGTGGAAAGTGGT 3’	5’ AAGCACTTGTAGGAAAAATTAAAATGA 3’	480
GLA-E7	5’ TTTTTCCTACAAGTGCTTGATAGTTCT 3’	5’ ACCTCAGGTGATCTGCCCG 3’	529

### Bioinformatics analysis

Bioinformatics analysis of the detected mutation was performed using Polyphen and SIFT software. The PolyPhen2 score ranges from 0 to 1, and the corresponding predictions are “probably damaging” (score > 0.85), “possibly damaging” (0.15 < score < 0.85), and “benign” (score < 0.15). If the SIFT score is smaller than 0.05, then the corresponding neutral substitutions (NS) are predicted to be “damaging”; if the SIFT score is greater than 0.05, then the NS are predicted as “tolerated”. The variant with a higher score is considered more deleterious in Phylop software.

### Site-directed mutagenesis of p.Asn278Lys in the *GLA* gene

Human alpha-Gal A (NM_000169.2) was amplified and cloned into the mammalian expression vector pcDNA3.1 (+) by introducing unique restriction sites for Hind III and Not I. The QuikChange II site-directed mutagenesis kit (Agilent Technologies, USA) was used for site-directed mutagenesis to generate a single nucleotide change (Asn278Lys, A to T) in the *GLA* gene sequence. Expression vectors harbouring the wild type (*GLA*-278Asn) or mutant (*GLA*-278Lys) sequences were identified by Sanger Sequencing.

### In vitro functional analysis of the p.Asn278Lys mutation in the *GLA* gene

HEK 293 T cells were cultured in 10% FBS high-glucose DMEM at 37 °C with 5% CO_2_ in a humidified incubator and seeded onto 24-well plates the day before transfection. Transient expression of the wild type and mutant enzymes was performed using Lipofectamine™ 2000 reagent (Invitrogen, USA) and antibiotic-free Opti-MEM® I Reduced Serum Medium (Gibco, Life Technologies, USA). After incubation for 4 h at 37 °C, the medium in the 24-well plates was replaced with 500 μl of fresh 10% FBS DMEM.

α-Gal A protein levels were detected by western blot analysis in cells expressing the wild type or variant *GLA* gene and the pharmacological chaperone 1-deoxy-galactonojirimycin (DGJ; Toronto Research Chemicals), a potent competitive inhibitor of α-Gal A. Briefly, HEK 293 T cells were lysed and centrifuged, and the supernatants were collected for analysis. A total of 50 μg of protein was separated on a 10% SDS-PAGE gel and then transferred to a PVDF membrane. After blocking with 5% non-fat milk, the membrane was incubated with primary antibodies against human α-Gal A (Santa Cruz Biotechnologies) overnight at 4 °C. After washing, the membranes were incubated for 2 h with the HRP-conjugated secondary antibody. The intensity of the bands was detected by using ECL and analysed with Gel Pro analysis software.

The enzyme activities of wild type and mutant α-Gal A were also investigated as previously reported [18, 19]. Briefly, the enzyme activity was calculated at pH 4.6 with 2 mM 4-MU-α-Gal (Sigma, Germany) as a substrate. The release of 4-MU per hour was determined by fluorescence measurement with an excitation wavelength of 360 nm and emission wavelength of 465 nm.

### Statistical analysis

Data analyses were conducted by SPSS 22.0 and GraphPad Prism5 software. Continuous variables are expressed as the means ± standard errors, and differences in enzyme activity and DGJ responsiveness between the wild type and mutant α-Gal A enzymes were assessed using Student’s *t* test.

## Results

### Clinical findings

**Fig. 1:**
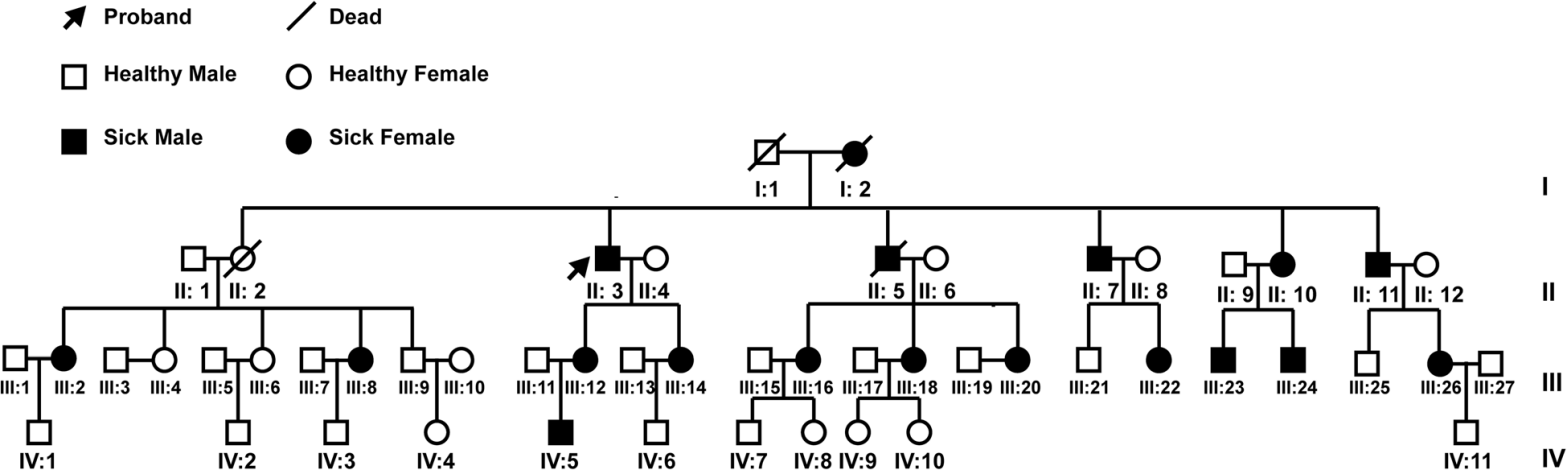
The four-generation pedigree with the mutation p. Asn278Lys in the *GLA* gene. Roman numerals indicate generations, and individuals within a generation are numbered from left to right. The proband (II: 3) is denoted with an arrow. Oblique lines indicate patients who are already dead. Filled squares and circles indicate male and female patients, respectively. Open symbols indicate unaffected individuals in this family

**Fig. 2:**
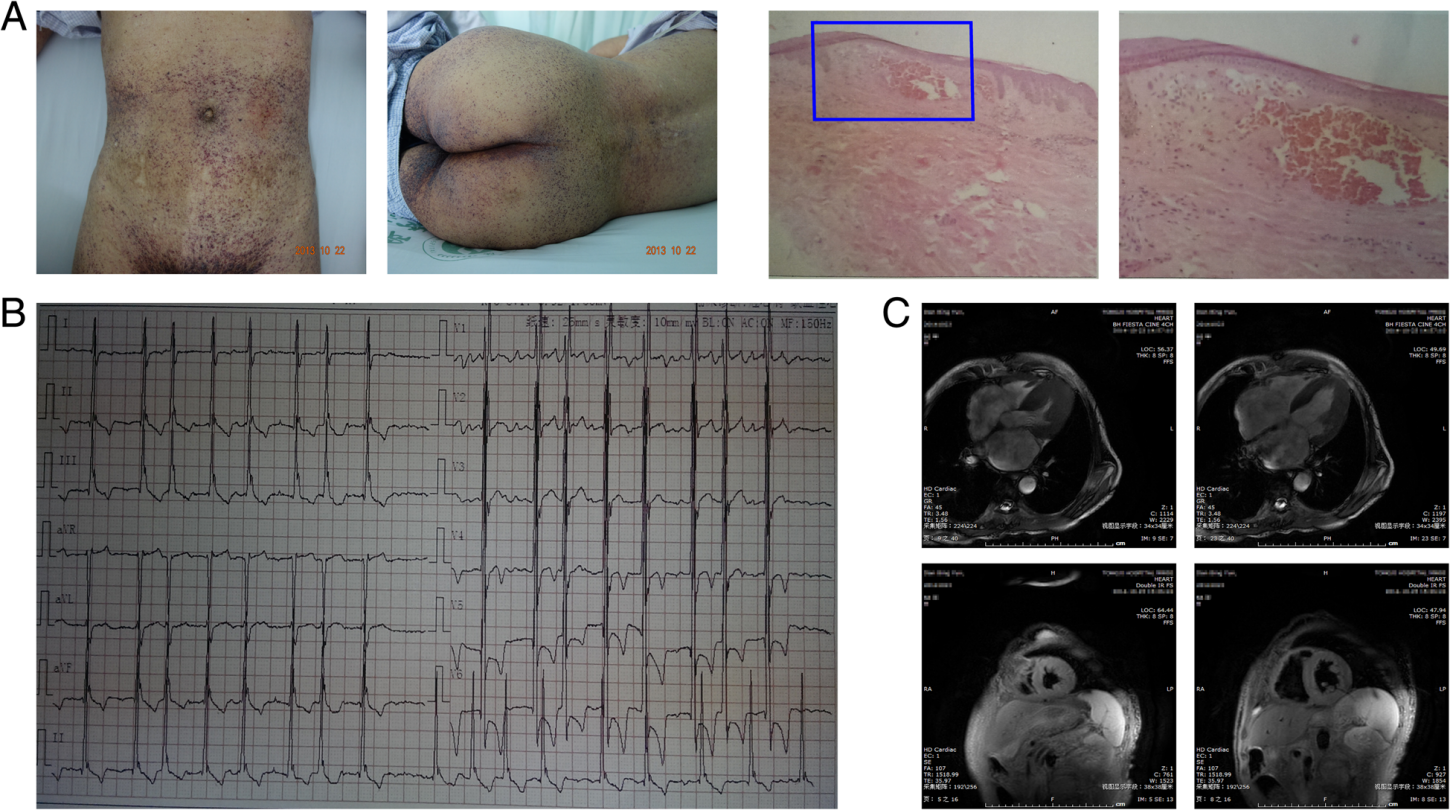
Clinical characteristics of the proband in this pedigree. **a** Pictures of skin rashes in the groin and on the back of the patient (Capillary balloons dilated on the surface of the skin are distributed as clusters or grape-like spots. The rash can be purplish red or red black when it is accompanied by venous bleeding, and excessive skin rash can be hyperkeratosis) and histopathology of skin biopsy (A wide range of crystallized sugar sphingomyelin deposits in the tissue can be observed under the microscope, and the histopathological diagnosis is angiokeratomas). **b** The electrocardiogram of the patient indicating atrial fibrillation rhythm, high voltage in the left ventricular, and T wave inversion in all precordial leads. **c** Cardiac magnetic resonance imaging showing apparent left ventricle hypertrophy at the apical region and the septum. The upper two pictures represent the sagittal plane of the heart, and the two lower figures show the coronal plane of the heart

The proband (II: 3), a 65-year-old male patient, was admitted as a cardiovascular inpatient 5 years ago due to chest tightness, dyspnoea (New York Heart Association functional class III) and suspected primary senile degenerative heart disease (Fig. 1). During the past 5 years, the patient received drug treatments, including vasodilators, diuretics, and cardiotonics, and the symptoms were slightly alleviated. Recalling his disease history, the patient displayed a three-year history of cerebral infarction, a one-year history of atrial fibrillation, a one-year history of thyroid dysfunction, and idiopathic thrombocytopenia for decades with several years of chronic diarrhoea. The patient also complained of frequent acroparaesthesia and hypohidrosis. Within 10 days prior to his visit to the hospital, the patient showed symptoms such as loss of appetite, bloating, abdominal pain, and vomiting after eating with no obvious trigger. The patient denied histories of hypertension, diabetes, and any infectious diseases. Physical examination of his skin showed a large area of skin rash that was particularly prominent in the groin and on the back, which were identified as angiokeratomas by histopathology (Fig. 2a). Blood tests showed mild anaemia (haemoglobin: 114 g/l), decreased eGFR (74.4 ml/min/1.73m^2^), an increased level of NT-proBNP (3904 pg/ml), and slightly elevated levels of troponin (0.046 ng/ml). Urine test results showed that the patient had proteinuria (1+). The electrocardiogram showed atrial fibrillation, voltage criteria for LV hypertrophy, and T wave inversion in all precordial leads (Fig. 2b). Transthoracic echocardiography demonstrated concentric LV hypertrophy (14 mm for the interventricular septum and 13 mm for the left ventricular posterior wall) and enlargement of the double atriums, consistent with signs detected by cardiac magnetic resonance imaging (Fig. 2c). Importantly, the enzyme activity of α-Gal A measured in the proband was very low, at 0.85 nmol/h/mg (> 30 nmol/h/mg for a normal person). A review of his family history revealed that two of his siblings (II: 2, female; and II: 5, male) presented renal dysfunction as the most severe manifestation and died from uraemia several years ago. His three surviving siblings (II: 7; II: 10; and II: 11), his two daughters (III: 12 and III: 14), nine of his nephews and nieces (III: 2; III: 8; III: 16; III: 18; III: 20: III: 22: III: 23: III: 24; and III: 26), and one of his grandsons (IV: 5) also had varying degrees of acroparaesthesia, hypohidrosis and chronic renal dysfunction. The male relatives exhibited more severe phenotypes than the female relatives. The clinical details of the subjects enrolled in this study are summarized in Table 2. The proband was treated based on the updated guidelines of heart failure. Specifically, vasodilators, diuretics, and digoxin were used to ameliorate the symptoms of heart failure, and a beta-blocker was used to improve the long-term prognosis. Warfarin was used to prevent stroke caused by atrial fibrillation. In addition, their families received genetic counselling in the genetic counselling centre of our hospital.

**Table 2:** Clinical characteristics of patients of the pedigree enrolled in this study

	Patients enrolled in this study						
	II: 3	II: 7	II: 10	II: 11	III: 12	III: 14	IV: 5
Age (y)	65	58	54	50	40	36	12
Sex	male	male	female	male	female	female	male
Neurological symptoms							
Neuropathic pain	**√**	**√**	/	**√**	/	/	/
Acroparaesthesia	**√**	**√**	/	**√**	/	/	**√**
Heat or cold intolerance	/	/	/	/	/	/	/
Fever crisis	/	/	/	/	/	/	/
Strokes	**√**	/	/	/	/	/	/
Seizures	/	/	/	/	/	/	/
Tegumentary symptoms							
Angiokeratoma	**√**	**√**	/	**√**	/	/	/
Hypohidrosis	**√**	**√**	/	**√**	/	/	**√**
Kidney symptoms							
Chronic renal dysfunction	**√**	**√**	**√**	**√**	**√**	**√**	/
Dialysis	/	**√**	/	**√**	/	/	/
Haematuria	/	N	N	N	N	N	N
Proteinuria	**√**	N	N	N	N	N	N
Cardiovascular symptoms							
Chest Pain	**√**	**√**	**√**	**√**	**√**	**√**	/
Dyspnoea	√	**√**	/	**√**	**√**	/	/
Syncope	/	/	/	/	/	/	/
Hypertension	/	**√**	/	/	/	/	/
Left ventricular hypertrophy	√	N	N	N	N	N	N
Ophthalmologic symptoms							
Corneal opacities	/	/	/	/	/	/	/
Gastrointestinal symptoms							
Nauseas	**√**	**√**	**√**	**√**	**√**	**√**	**√**
Abdominal Pain	**√**	**√**	/	**√**	/	/	**√**
Chronic diarrhoea	**√**	**√**	**√**	**√**	**√**	**√**	**√**
Metabolic symptoms							
Dyslipidaemia	/	/	/	/	/	/	/
Genotype of p. Asn278Lys site	X^b^Y	X^b^Y	X^B^X^b^	X^b^Y	X^B^X^b^	X^B^X^b^	X^b^Y

“B” represents the wild type genotype (*GLA*-Asn278), and “b” represents the mutant genotype (*GLA*-Lys278)

### Genetic screening and functional identification

**Fig. 3:**
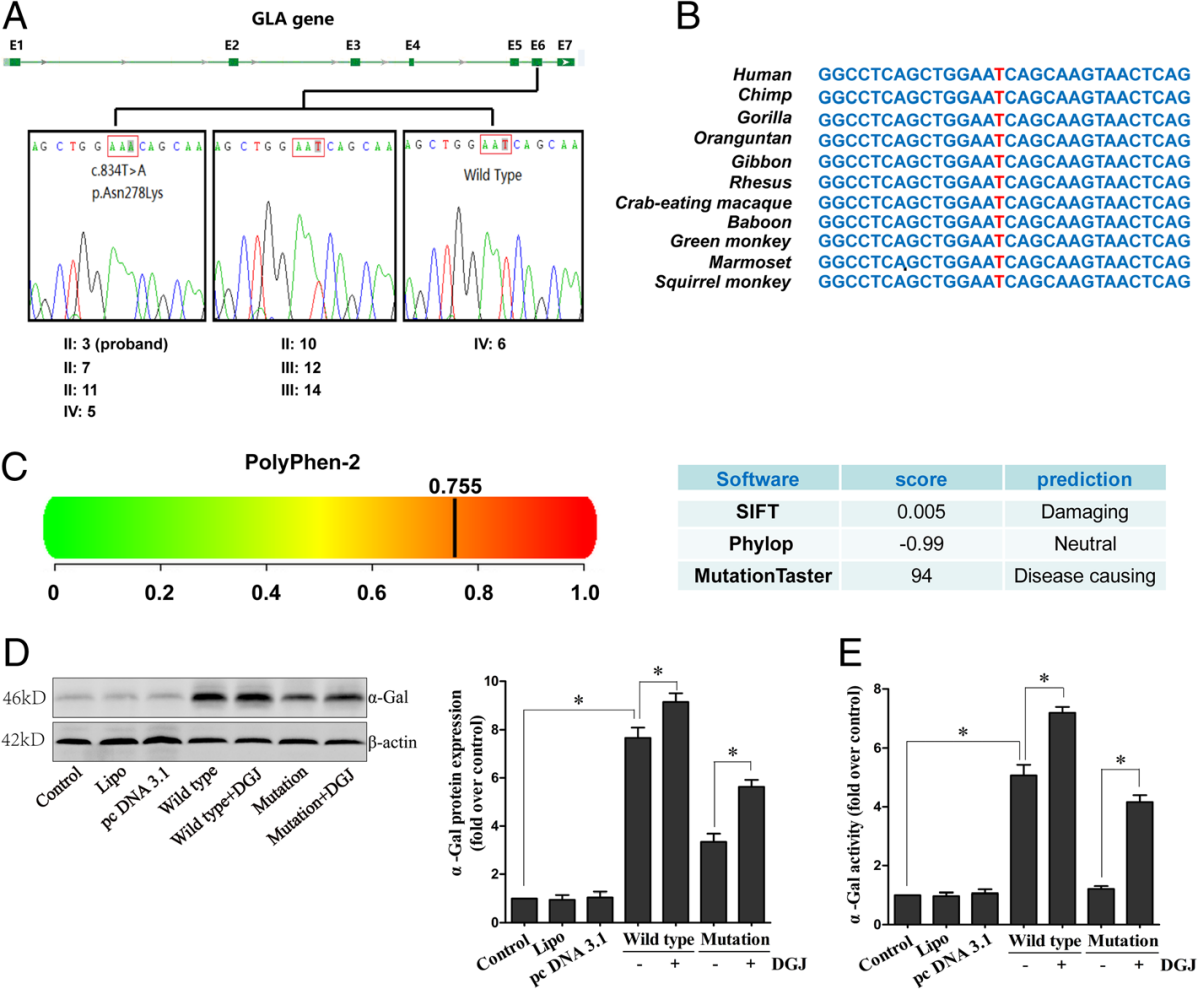
Genetic screening and functional identification. **a** DNA sequences of the p.Asn278Lys site and adjacent bases of the eight subjects enrolled in the present study. **b** Genome Browser database of vertebrate species at the p.Asn278Lys mutation site and surrounding sequences. **c** In silico bioinformatic predictions for the function of the p.Asn278Lys mutation were made by four independent software packages: Polyphen-2, SIFT, Phylop, and MutationTaster. **d** Representative immunoblots and quantitation of α-Gal A protein expression with or without DGJ treatment in HEK293T cell lines transfected with GLA-Asn278 or GLA-Lys278. **e** Measurements of α-Gal A enzyme activity with or without DGJ treatment in KEK293T cell lines transfected with GLA-Asn278 or GLA-Lys278. Data are expressed as the means ± SD, *n* = 3 for each group, *p < 0.05

Detailed physical examination and laboratory inspections strongly conflicted with the previous diagnosis and suggested FD. To further clarify the diagnosis, we screened the entire coding region of the *GLA* gene for the proband of this pedigree and detected no mutations, except for p. Asn278Lys (c. 834 T > A) in exon 6 (Fig. 3a). We obtained blood samples from his three siblings (II-7, II-10 and II-11), two daughters (III-12 and III-14) and two grandsons (IV-5 and IV-6) and genotyped this mutation site by sequencing. The hemizygous p. Asn278Lys mutation was identified in the 3 male patients (II-7, II-11 and IV-5), and the heterozygous p. Asn278Lys mutation was identified in the 3 female patients (II-10, III-12, and III-14) in this pedigree. IV-6 (grandson of the proband) showed no abnormal clinical manifestations and was shown to be wild type at this site by sequencing.

The Genome Browser database (http://genome.ucsc.edu) of vertebrate species revealed a strictly conserved genomic structure around the mutation site among vertebrate species (Fig. 3b). The function of the novel c.834 T > A mutation (p. Asn278Lys) was evaluated in silico by 4 independent software programs at the protein level. This mutation is predicted to be “possibly damaging” with a score of 0.755 by Polyphen-2. MutationTaster software annotated this mutation as “disease-causing” with a prediction score of 94. SIFT predicted this variant as a “damaging variant” (SIFT score = 0.005), while Phylop treated this mutation as a neutral variant with a score of − 0.99 (Fig. 3c). In addition, this mutation was shown to be absent in a control cohort of 300 local people.

To further identify the biological characteristics of the mutant enzyme in terms of protein degradation and activity, we developed an over-expression system in HEK-293 T cells to investigate the in vitro characteristics of the wild and mutant enzymes. When 293 T cells were transfected with *GLA*-Asn278 or *GLA*-Lys278, we obtained successful α-Gal A expression compared with the group transfected with the empty pcDNA3.1(+) vector. However, the mutant α-Gal A protein exhibited lower levels than the wild type protein (Fig. 3d). Similar results were observed for the residual activity of the mutant enzyme. As shown in Fig. 3e, the enzyme activity of α-Gal A was significantly lower in cells transfected with *GLA*-Lys278 compared with *GLA*-Asn278 (Fig. 3e).

To evaluate the therapeutic effect of DGJ as a potential drug for this particular mutation, we analysed the protein expression of α-GLA and enzyme activity changes after DGJ treatment. The results showed that the pharmacological chaperone DGJ increased the expression of α-Gal (Fig. 3d) and markedly normalized the activity of this enzyme (Fig. 3e), especially in cells expressing *GLA*-Lys278, which indicated that patients possessing this specific mutation were good responders to the potential biochemical therapy.

## Discussion

FD is an X-linked recessive lysosomal storage disorder caused by deficiency of the enzyme α-Gal A secondary to *GLA* gene mutations. In this study, we reported a large four-generation Chinese pedigree diagnosed with FD by whole-exon sequencing of the *GLA* gene. We identified a novel mutation (loss-of-function) in the *GLA* gene, p.Asn278Lys, by PCR-direct sequencing, co-segregation analysis and functional evaluation of this mutation in an over expression cell system. We first demonstrated that the substitution of lysine for asparagine at residue 278 of the amino acid sequence of the *GLA* gene caused a decrease in the expression level of the α-Gal A protein as well as the activity of this enzyme. In addition, this enzyme deficiency was effectively normalized by the pharmacological chaperone DGJ, a competitive inhibitor of α-Gal A and a potential therapeutic agent for patients with FD with amenable mutations.

FD affects multiple systems throughout the body, and the clinical spectrum varies among different individuals. In the past, FD was only detected in hemizygous male patients due to its X-linked transmission, and female heterozygous patients were assumed to be only carriers. However, male patients can also have atypical presentations, and female heterozygotes can develop severe clinical manifestations, including chronic kidney disease, and disorders of the heart and nervous systems. Therefore, it is more precise to integrally define FD as a disorder with classical and atypical phenotypes. Patients diagnosed with the classic subtype show multi-organ dysfunctions, such as arrhythmia, heart failure, chronic kidney disease and acroparaesthesia. Additionally, the atypical or late-onset subtype may be characterized by single organ involvement, most of which present as cardiac and renal dysfunction in late life. Further studies have shown that the function of the *GLA* gene is completely lost in the classic subtype but is partially retained in the variant types. This finding is coincident with the manifestations of the two subtypes [20]. The proband of this pedigree did not develop classic symptoms during childhood, and his prominent symptoms were atrial fibrillation, cardiac hypertrophy, heart failure, and angiokeratoma for many years. He received treatment for heart failure, but the response was only partial. The patients of this family did not present with the classic phenotype, and the involved organs and predominant symptoms were inconsistent, further complicating diagnosis. Genetic testing revealed the mutation p.Asn278Lys, which was consistent with the phenotype of this family. As bioinformatics predictions are controversial, we additionally identified the function of this mutation as a loss-of-function mutation in an over-expression cell system. We identified the genetic variant among the family members, which could guide a thorough change in the therapeutic strategy and prognosis of future offspring of this family.

ERT has widespread therapeutic efficacy in FD, which is a rare X-linked inherited disease [12, 13]. ERT has been available since 2001, and two recombinant enzyme preparations, agalsidase alpha and agalsidase beta, have been approved for FD treatment. However, some deficiencies limit the use of this therapeutic strategy. First, once severe organ damage has occurred, such as in the heart or kidney, ERT cannot always achieve the expected ideal effect [21, 22]. Second, recombinant enzyme administration typically induces the generation of anti-α-Gal A antibodies [23]. Third, ERT requires lifelong treatment, resulting in huge medical expenses [24]. Another promising therapy is the pharmacological chaperone DGJ, which is a potent α-Gal A inhibitor that can promote the proper folding of mutated enzymes by binding to its active site [25]. Previous studies have indicated that DGJ seems to prevent the early degradation of the α-Gal enzyme in the endoplasmic reticulum and accelerates the maturation of the mutant enzyme [18, 26]. Phase II clinical trials showed that DGJ was safe and well-tolerated, and a phase III clinical trial is underway in the USA [25]. Migalastat, an oral pharmacological chaperone, stabilizes specific mutant forms of α-galactosidase, to facilitate enzyme trafficking to lysosomes [27–29]. The mutation detected in this Chinese pedigree (p. Asn278Lys) showed a good response to DGJ, and the protein expression of α-Gal was significantly increased in cells transfected with a plasmid encoding mutant GLA. Thus, patients with this specific mutation may be suitable for the application of DGJ therapy. It is well-established that viral vector-mediated gene therapy is a potential biotherapy for Fabry mice [30]. Myocardial morphology and function can be improved in the long-term if the therapy is initiated prior to the development of myocardial fibrosis.

Given the potential benefits of early medical and psychological interventions, genetic counselling and evaluation are extremely important and strongly recommended for those suspected cases. Hundreds of mutations have been associated with FD, but the clinical significance of these mutations has not been well clarified. Previous studies have investigated the association of a specific mutation with the phenotype of FD and responses to DGJ. The results showed that most of these mutations are associated with variant and mild classic phenotypes (with residual α-Gal-A activity), and 42.8% of the missense mutants were responsive to DGJ [18, 31], suggesting that DGJ treatment may be widely applicable to FD with some specific amenable mutations [32]. However, the strengths and weaknesses of the different treatments and routes of administration require further discussion and evaluation in future studies.

## Conclusions

In this study, we identified a novel *GLA* p. Asn278Lys mutation in a four-generation Chinese pedigree with FD and validated its biological function (loss-of-function) and response to the pharmacological chaperone DGJ in an over-expression cell system. In recent years, the diagnosis of FD has gradually progressed, and the underlying genetic mechanism is becoming well-defined. Future studies should focus on therapeutic interventions to significantly improve patient quality of life.

## Abbreviations

DGJ: 1-deoxy-galactonojirimycin
ERT: Enzyme replacement therapy
FD: Fabry disease
NS: Neutral substitutions
α-Gal A: α-galactosidase A

## References

[1] Fabry J. Ein Beitrag zur Kenntnis der Purpura haemorrhagica nodularis (Purpura haemorrhagica Hebrae). Arch Dermatol Syph. 1898;43(1):187–200.

[2] Anderson W. A case of angiokeratoma. Br J Dermatol. 1898;10:113–7.

[3] Garman SC, Garboczi DN (2004). The molecular defect leading to Fabry disease: structure of human alpha-galactosidase. J Mol Biol.

[4] Anastasakis A, Sevdalis E, Papatheodorou E, Stefanadis C (2011). Anderson-Fabry disease: a cardiomyopathy that can be cured. Hellenic J Cardiol.

[5] Lidove O, Kaminsky P, Hachulla E, Leguy-Seguin V, Lavigne C, Marie I, Maillot F, Serratrice C, Masseau A, Cherin P (2012). Fabry disease 'The new great Imposter': results of the French Observatoire in Internal Medicine Departments (FIMeD). Clin Genet.

[6] Marchesoni CL, Roa N, Pardal AM, Neumann P, Caceres G, Martinez P, Kisinovsky I, Bianchi S, Tarabuso AL, Reisin RC (2010). Misdiagnosis in Fabry disease. J Pediatr.

[7] Toyooka K (2011). Fabry disease. Curr Opin Neurol.

[8] Thomas AS, Hughes DA (2014). Fabry disease. Pediatr Endocrinol Rev.

[9] Germain DP (2010). Fabry disease. Orphanet J Rare Dis.

[10] Duro G, Musumeci MB, Colomba P, Zizzo C, Albeggiani G, Mastromarino V, Volpe M, Autore C (2014). Novel alpha-galactosidase a mutation in patients with severe cardiac manifestations of Fabry disease. Gene.

[11] Peng H, Xu X, Zhang L, Zhang X, Peng H, Zheng Y, Luo S, Guo H, Xia K, Li J (2016). GLA variation p.E66Q identified as the genetic etiology of Fabry disease using exome sequencing. Gene.

[12] Schiffmann R, Kopp JB, Austin HA, Sabnis S, Moore DF, Weibel T, Balow JE, Brady RO (2001). Enzyme replacement therapy in Fabry disease: a randomized controlled trial. Jama.

[13] Eng CM, Guffon N, Wilcox WR, Germain DP, Lee P, Waldek S, Caplan L, Linthorst GE, Desnick RJ, International collaborative Fabry disease study G (2001). Safety and efficacy of recombinant human alpha-galactosidase a replacement therapy in Fabry's disease. N Engl J Med.

[14] Parini R, Schiffmann R, Fotheringham I, Todorova L (2015). A systematic review of the humanistic burden of disease in patients with Fabry disease. Value Health.

[15] Kalliokoski RJ, Kantola I, Kalliokoski KK, Engblom E, Sundell J, Hannukainen JC, Janatuinen T, Raitakari OT, Knuuti J, Penttinen M (2006). The effect of 12-month enzyme replacement therapy on myocardial perfusion in patients with Fabry disease. J Inherit Metab Dis.

[16] Koskenvuo JW, Hartiala JJ, Nuutila P, Kalliokoski R, Viikari JS, Engblom E, Penttinen M, Knuuti J, Mononen I, Kantola IM (2008). Twenty-four-month alpha-galactosidase a replacement therapy in Fabry disease has only minimal effects on symptoms and cardiovascular parameters. J Inherit Metab Dis.

[17] Mursa A, Ginghina C, Jurcut R (2014). Fabry disease--a primer for cardiologists. Rom J Intern Med.

[18] Lukas J, Giese AK, Markoff A, Grittner U, Kolodny E, Mascher H, Lackner KJ, Meyer W, Wree P, Saviouk V (2013). Functional characterisation of alpha-galactosidase a mutations as a basis for a new classification system in fabry disease. PLoS Genet.

[19] Lukas J, Scalia S, Eichler S, Pockrandt AM, Dehn N, Cozma C, Giese AK, Rolfs A (2016). Functional and clinical consequences of novel alpha-galactosidase a mutations in Fabry disease. Hum Mutat.

[20] Nakao S, Kodama C, Takenaka T, Tanaka A, Yasumoto Y, Yoshida A, Kanzaki T, Enriquez AL, Eng CM, Tanaka H (2003). Fabry disease: detection of undiagnosed hemodialysis patients and identification of a "renal variant" phenotype. Kidney Int.

[21] Weidemann F, Niemann M, Breunig F, Herrmann S, Beer M, Stork S, Voelker W, Ertl G, Wanner C, Strotmann J (2009). Long-term effects of enzyme replacement therapy on fabry cardiomyopathy: evidence for a better outcome with early treatment. Circulation.

[22] Warnock DG, Ortiz A, Mauer M, Linthorst GE, Oliveira JP, Serra AL, Marodi L, Mignani R, Vujkovac B, Beitner-Johnson D (2012). Renal outcomes of agalsidase beta treatment for Fabry disease: role of proteinuria and timing of treatment initiation. Nephrol Dial Transplant.

[23] Wilcox WR, Linthorst GE, Germain DP, Feldt-Rasmussen U, Waldek S, Richards SM, Beitner-Johnson D, Cizmarik M, Cole JA, Kingma W (2012). Anti-alpha-galactosidase a antibody response to agalsidase beta treatment: data from the Fabry registry. Mol Genet Metab.

[24] Vedder AC, Linthorst GE, Houge G, Groener JE, Ormel EE, Bouma BJ, Aerts JM, Hirth A, Hollak CE (2007). Treatment of Fabry disease: outcome of a comparative trial with agalsidase alfa or beta at a dose of 0.2 mg/kg. PLoS One.

[25] Ishii S (2012). Pharmacological chaperone therapy for Fabry disease. Proc Jpn Acad Ser B Phys Biol Sci.

[26] Fan JQ, Ishii S, Asano N, Suzuki Y (1999). Accelerated transport and maturation of lysosomal alpha-galactosidase a in Fabry lymphoblasts by an enzyme inhibitor. Nat Med.

[27] Hughes DA, Nicholls K, Shankar SP, Sunder-Plassmann G, Koeller D, Nedd K, Vockley G, Hamazaki T, Lachmann R, Ohashi T (2017). Oral pharmacological chaperone migalastat compared with enzyme replacement therapy in Fabry disease: 18-month results from the randomised phase III ATTRACT study. J Med Genet.

[28] Benjamin ER, Della Valle MC, Wu X, Katz E, Pruthi F, Bond S, Bronfin B, Williams H, Yu J, Bichet DG (2017). The validation of pharmacogenetics for the identification of Fabry patients to be treated with migalastat. Genet Med.

[29] Germain DP, Hughes DA, Nicholls K, Bichet DG, Giugliani R, Wilcox WR, Feliciani C, Shankar SP, Ezgu F, Amartino H (2016). Treatment of Fabry's disease with the pharmacologic chaperone Migalastat. N Engl J Med.

[30] Ruiz de Garibay AP, Solinis MA, Rodriguez-Gascon A (2013). Gene therapy for fabry disease: a review of the literature. BioDrugs.

[31] Ishii S, Chang HH, Kawasaki K, Yasuda K, Wu HL, Garman SC, Fan JQ (2007). Mutant alpha-galactosidase a enzymes identified in Fabry disease patients with residual enzyme activity: biochemical characterization and restoration of normal intracellular processing by 1-deoxygalactonojirimycin. The Biochemical journal.

[32] Siekierska A, De Baets G, Reumers J, Gallardo R, Rudyak S, Broersen K, Couceiro J, Van Durme J, Schymkowitz J, Rousseau F (2012). Alpha-galactosidase aggregation is a determinant of pharmacological chaperone efficacy on Fabry disease mutants. J Biol Chem.

